# Ring the BELL and tie the KNOX: roles for TALEs in gynoecium development

**DOI:** 10.3389/fpls.2014.00093

**Published:** 2014-03-20

**Authors:** Nicolas Arnaud, Véronique Pautot

**Affiliations:** UMR 1318 INRA-AgroParisTech, INRA Centre de Versailles-Grignon, Institut Jean-Pierre BourginVersailles, France

**Keywords:** carpel, TALE, transcription factors, development, *Arabidopsis*

## Abstract

Carpels are leaf-like structures that bear ovules, and thus play a crucial role in the plant life cycle. In angiosperms, carpels are the last organs produced by the floral meristem and they differentiate a specialized meristematic tissue from which ovules develop. Members of the three-amino-acid-loop-extension (TALE) class of homeoproteins constitute major regulators of meristematic activity. This family contains KNOTTED-like (KNOX) and BEL1-like (BLH or BELL) homeodomain proteins, which function as heterodimers. KNOX proteins can have different BELL partners, leading to multiple combinations with distinct activities, and thus regulate many aspects of plant morphogenesis, including gynoecium development. TALE proteins act primarily through direct regulation of hormonal pathways and key transcriptional regulators. This review focuses on the contribution of TALE proteins to gynoecium development and connects TALE transcription factors to carpel gene regulatory networks.

## INTRODUCTION

**FIGURE 1:**
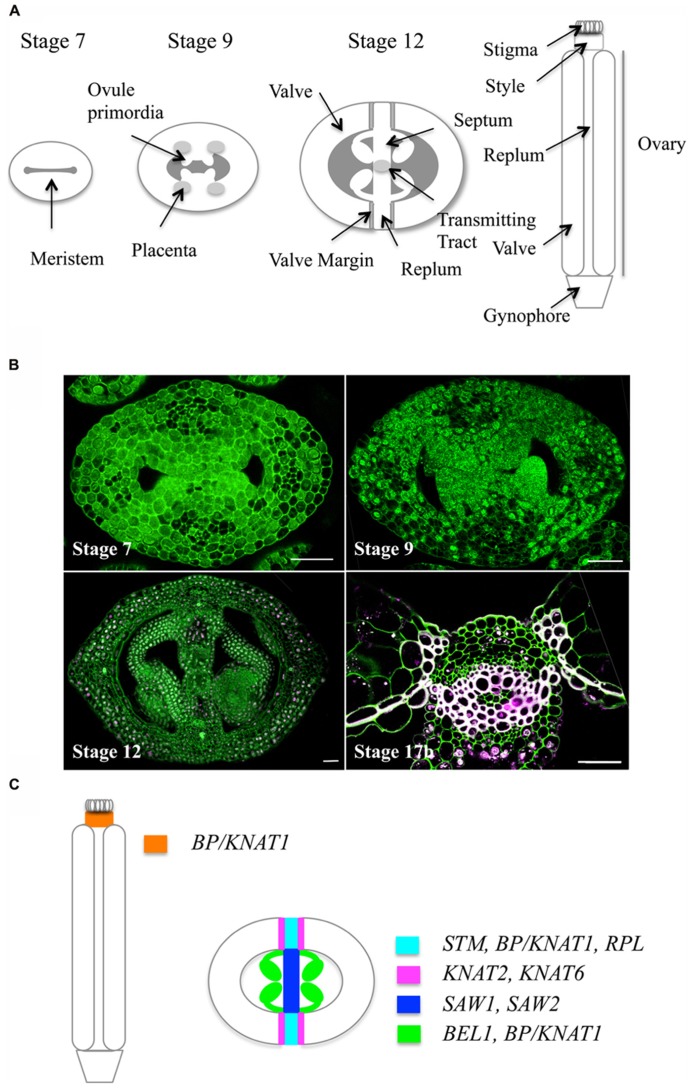
***Arabidopsis* gynoecium development.** **(A)** Schematic cross sections showing the different tissues of the gynoecium at three developmental stages according to Smyth et al. (1990). **(B)** Optical cross sections through the *Arabidopsis* gynoecium at four developmental stages stained with iodine green and carmine alum: upper left, stage 7, showing the layered structure of the meristem; upper right, stage 9, showing ovule primordia initiating from the placenta; lower left, stage 12, lower left, close-up of the medial tissue (stage 17b) showing the replum and lignin deposition at the valve margins and at the endocarp b layer. Scale bars represent 25 μm. **(C)** Schematic representation of expression patterns of *TALE* genes in the *Arabidopsis* gynoecium (stage 12).

In *Arabidopsis*, the female reproductive organ, or gynoecium, consists of an apical stigma, a style, and a basal ovary (**Figure 1** and for reviews, Ferrándiz et al., 1999; Roeder and Yanofsky, 2006; Girin et al., 2009; Ferrándiz et al., 2010). The ovary is composed of two fused carpels (termed valves after fertilization) whose margins are joined by the replum. The inner (adaxial) side of the replum has a typical meristematic layered structure. This meristem gives rise to ovules and to two septum primordia, which grow and fuse to create the septum that divides the ovary into two locules. Two rows of ovules arise along the septum inside each locule. The septum differentiates a central transmitting tract tissue, which guides pollen tubes from the style to the ovule. Upon fertilization, ovules develop into seeds, and gynoecium structure changes dramatically: the fruit enlarges both longitudinally and laterally to accommodate seed growth and the valve margins undergo cell wall changes required for silique dehiscence and seed dispersal.

In multicellular organisms, development relies on stem cells, which are defined by their ability to renew themselves and to give rise to daughter cells that contribute to organ production. In plants, stem cells are maintained within structures called meristems, and new organs are produced at the meristem periphery (for review, Sablowski, 2011). The shoot apical meristem (SAM) produces leaves and axillary meristems. Following floral evocation, the SAM becomes an inflorescence meristem (IM), which produces flower meristems (FMs) that give rise to flowers containing gynoecia. Carpels are thought to be modified leaves with their margins representing a lateral organ boundary (Frohlich, 2003). As such, similar interactions occurring between SAM-boundary-leaf apply to fruit patterning.

Within meristems, cell proliferation and differentiation are tightly controlled by networks of transcription factors (TFs), which integrate developmental cues such as position, differentiation, and growth (Sablowski, 2011). The *KNOTTED1* (*KN1*) gene in maize was the first regulator of meristem activity identified in plants (Hake and Vollbrecht, 1989). In *Arabidopsis*, *SHOOT MERISTEMLESS* (*STM*), which is functionally related to *KN1,* and *WUSCHEL* (*WUS*) control meristem activity (for review, Aichinger et al., 2012). *WUS* is required to maintain the stem-cell population, as *wus* mutants lack stem cells at the center of the shoot apices while *STM* is required for SAM initiation and its maintenance in an undifferentiated state, as strong *stm* mutants fail to develop a meristem during embryogenesis and fail to produce lateral organs (Endrizzi et al., 1996; Long et al., 1996). *STM* is expressed in SAM, IM, FM, and in the inner side of the replum (Endrizzi et al., 1996; Long et al., 1996; Ragni et al., 2008). *STM* is down-regulated when cells become specified as primordium founder cells (Long et al., 1996).

STM belongs to the “Three-Amino-acid-Loop-Extension” (TALE) homeodomain superclass of TFs, which in *Arabidopsis* comprises 9 KNOTTED-like (KNAT or KNOX) and 13 BEL1-like (BLH or BELL) members (**Box 1**). The TALE factors function as KNOX-BELL heterodimers (for reviews, Hay and Tsiantis, 2010; Hamant and Pautot, 2010; Di Giacomo et al., 2013). STM maintains the pool of indeterminate meristematic cells through repression of gibberellin (GA) biosynthesis, activation of GA catabolism, and activation of cytokinin (CK) biosynthesis (Sakamoto et al., 2001; Chen et al., 2004; Jasinski et al., 2005; Bolduc and Hake, 2009). In addition, in the SAM, STM represses the *ASYMMETRIC LEAVES1* (*AS1*) gene, which encodes a MYB TF involved in leaf patterning. AS1 represses other TALE-family members such as *KNAT1/BREVIPEDICELLUS* (*BP*), *KNAT2*, and *KNAT6* in leaves (Byrne et al., 2000; Phelps-Durr et al., 2005). Subsequent organ initiation requires high auxin and GA levels and down regulation of *STM* and related TFs (Hay and Tsiantis, 2010).

**Box 1. Meet the TALE gene family**

The TALE family is a superclass of homeodomain TFs which comprises eight KNOTTED-like proteins in *Arabidopsis thaliana* (KNAT or KNOX) plus a mini KNAT lacking the homeodomain (KNATM) and 13 BEL1-like (BLH or BELL) members (for a detailed review of the structure of this gene family, see Mukherjee et al. (2009) and for a phylogenetic tree of the *Arabidopsis* TALE family, see Hamant and Pautot, 2010). This family controls development in all eukaryotic lineages (Hay and Tsiantis, 2010). KNOX and BELL families occur in single copy in Green algae and have diversified in land plant (Lee et al., 2008). The KNOX family is divided into three classes based on sequence similarity and gene expression pattern (Hake et al., 2004; Magnani and Hake, 2008): Class I includes STM, BP/KNAT1, KNAT2 and KNAT6; Class II includes KNAT3, 4, 5, and 7. Class III contains KNATM, which can interact with other TALE members to modulate their activity (Kimura et al., 2008; Magnani and Hake, 2008). The BELL family comprises RPL/BLH9, PNF/BLH8, ATH1, SAW1/BLH2, SAW2/BLH8, BEL1 – whose functions have been characterized- and BLH1, BLH3, BLH5, BLH6, BLH7, BLH10, and BLH11 – whose functions are not yet known (Hamant and Pautot, 2010). The interaction of KNOX and BELL proteins is critical for their nuclear localization and their binding affinity to DNA, thereby imparting their activity (Smith et al., 2002; Rutjens et al., 2009; Kim et al., 2013). TALEs can also form complexes with other TFs, such as MADS-Box family members, to control ovule development (Brambilla et al., 2007) and with OVATE proteins, which negatively regulate KNOX-BELL heterodimers by relocalizing them from the nucleus to the cytoplasm (Hackbusch et al., 2005). STM protein traffics selectively through plasmodesmata, and this cell-to-cell movement, which involves chaperonins belonging to a group of cytosolic chaperones, is critical to maintain of the SAM (Xu et al., 2011).

Multiple combinations of TALE heterodimers with distinct activities are produced throughout the plant life cycle, controlling diverse developmental processes, such as SAM and boundary maintenance, leaf development and flowering. This review discusses the contribution of TALE TFs to gynoecium development in *Arabidopsis*, and links these proteins to the other key molecular players of carpel development.

### CARPEL INITIATION: KNOX AND BELL INTERACTIONS WITH AGAMOUS

Carpels are the last organs to be produced by floral meristems. Weak *STM* alleles or weak *STM* RNAi lines show no carpel formation due to premature differentiation of meristematic cells (Endrizzi et al., 1996; Scofield et al., 2007). Consistent with this, CLAVATA (CLV) receptors control the proliferation and number of organs in developing gynoecia through STM activity. Mutations in CLV1, CLV2, and CORYNE (CRN) receptors lead to increased meristem size correlated with an enlarged *STM* expression pattern (Durbak and Tax, 2011). Unlike the SAM, which is indeterminate, the FM terminates after carpel initiation. This determinacy depends on a negative feedback loop involving the C-function homeotic MADS domain TF, AGAMOUS (AG) which acts in part via activation of the zinc finger protein KNUCKLES (KNU) to repress *WUS* expression (Lenhard et al., 2001; Lohmann et al., 2001; Sun et al., 2009).****AG controls carpel identity in combination with another MADS BOX TF, SEPALLATA3 (SEP3)****(Bowman et al., 1989; Honma and Goto, 2001; Pelaz et al., 2001). *AG* expression is first detectable in developing flowers at early stage 3, flower stages defined by Smyth et al. (1990), where it is initially localized in the center of the FM, and is later restricted to stamen and carpel primordia (Bowman et al., 1991; Drews et al., 1991). At late stage 5, the floral meristem forms a flattened oval where the gynoecium initiates (Smyth et al., 1990). This stage coincides presumably with the generation of auxin maxima similar to those observed at the initiation of other organs, although no expression of auxin-signaling reporters at stages 5–7 has been described (for review, Larsson et al., 2013). The BELL member, REPLUMLESS (RPL), also known as PENNYWISE (PNY), BELLRINGER (BLR), VAAMANA (VAN), or LARSON (LSN) and its close relative POUNDFOOLISH (PNF) together with STM, function in parallel with LEAFY (LFY) and WUS to promote carpel formation through positive regulation of *AG *(Byrne et al., 2003; Roeder et al., 2003; Smith and Hake, 2003; Bao et al., 2004; Bhatt et al., 2004; Yu et al., 2009). Interestingly, a previous report showed that RPL represses *AG* together with LEUNIG and SEUSS, two transcriptional co-regulators of AG (Bao et al., 2004). This study was based on analysis of two recessive *rpl* alleles (*blr-4* and *blr-5*) whose flowers exhibit homeotic conversion of sepals to carpels at high temperature during late-stage flower development. This suggests that RPL could have two antagonistic activities depending presumably on its partner. However, no ectopic *AG* expression has been reported so far in null *rpl* mutants. An alternative hypothesis is that the point mutations within the homeobox region in *blr-4* and *blr-5* mutants cause the production of abnormal protein with regulatory defects.

### GYNOECIUM PATTERNING

Once initiated, the gynoecium developmental program promotes correct patterning of the future fruit. Several specific tissues are formed (see above and **Figure 1**), some of which require the activity of TALE TFs. At stage 6, the gynoecium forms as a ridge of raised cells around a central cleft and starts to acquire its medio-lateral symmetry, comprising replum, valve margins and valves. In the transverse plane, the adaxial inner side of the replum has a typical meristematic layered structure (**Figure 1B**), and accordingly expresses the meristematic genes *STM, CLV1/2*,and* CRN *(Long et al., 1996; Durbak and Tax, 2011; Romera-Branchat et al., 2012).However*, WUS *is not expressed in the replum(Groß–Hardt et al.,2002). Recently, a role for* WUS-LIKE HOMEOBOX13 (WOX13) *in replum was reported (Romera-Branchat et al., 2012). Unlike *WUS*, which marks a few cells in the SAM, defining its organizing center, *WOX13* has a broad expression pattern in replum, suggesting that the medial region of the gynoecium does not show typical SAM organization.

Consistent with a role for *STM* in initiating and maintaining meristems, weak alleles of *STM* or weak *STM* RNAi lines produce fewer ovules than the wild type (Endrizzi et al., 1996; Scofield et al., 2007). Two other TALE genes, *RPL* and *BP*, are also expressed in the replum. The *rpl* mutant shows defects in replum differentiation and in septum fusion (Roeder et al., 2003). RPL promotes replum identity through restriction of expression of the MADS-BOX genes *SHATTERPROOF1/2* (*SHP1/2*) and the basic helix-loop-helix (bHLH) gene *INDEHISCENT *(*IND*), to the valve margins****(Roeder et al., 2003; Liljegren et al., 2004; Dinneny et al., 2005), but RPL is not required *per se* for replum specification, since double or triple mutant combinations including *rpl *alleles develop a normal replum. In addition, RPL represses several valves-associated genes in the replum: *JAGGED* (*JAG*), and genes conferring abaxial fate *FILAMENTOUS FLOWER* (*FIL*)****and*****YABBY3* (*YAB3*), which promote *FRUITFULL* (*FUL*), *SHP1*/*2* and *IND* expression in the presumptive valve and valve margin tissues, respectively (Dinneny et al., 2005). BP, which interacts with RPL and activates its expression, contributes redundantly with RPL to replum development (Alonso-Cantabrana et al., 2007). Similarly to their role at the leaf/SAM interface, AS1 and the lateral organ boundary (LOB)-domain protein asymmetric leaves2 (AS2) restrict the expression of *BP* to the replum, exemplifying the co-option of this regulatory module in the SAM and carpel (Alonso-Cantabrana et al., 2007; González-Reig et al., 2012; Luo et al., 2012; Lodha et al., 2013). Together these studies led to proposition of a model in which antagonism between the lateral factors (JAG/FIL and AS1/2) and the medial factors (BP and RPL) determines the medio-lateral fruit pattern by regulating the formation and size of three domains: valve, valve margin and replum. Furthermore, APETALA2, a member of the AP2/Ethylene-responsive element binding protein (EREBP) TF family, limits growth of both replum and valve margins by repressing *BP* and *RPL* in the replum and *SHP1*/*2* and *IND* in valve margins (Ripoll et al., 2011). Although *BP*, together with *RPL*, contributes to replum development, single *bp* loss-of-function mutants have wild-type repla (Alonso-Cantabrana et al., 2007; Ripoll et al., 2011). BP is also expressed in the style where it is required for radial growth (Venglat et al., 2002).

From the maternal side, optimal seed production relies on adequate generation of ovules. Ovule primordia formation depends on auxin maxima (Bencivenga et al., 2012). Auxin levels are modulated by the combined activity of CUP-SHAPED COTYLEDON1 (CUC1) and CUC2 TFs, which are redundantly required to regulate the polar auxin transporter *pin-formed1 *(*PIN1*) expression (Galbiati et al., 2013). CK also regulates *PIN1* expression during early stages of ovule development (Bencivenga et al., 2012). The interplay between hormones and TFs forms an integrative framework enabling ovule primordia initiation. Once initiated, an ovule differentiates a central nucellus containing the embryo sac, two integuments that envelop the nucellus, and a funiculus that connects the ovule to the placenta (for reviews, Colombo et al., 2008; Shi and Yang, 2011). Correct ovule development requires the activity of the *BEL1* gene, the founding member of the BELL family. *BEL1* is expressed in ovule integument primordia, and controls ovule integument identity (Robinson-Beers et al., 1992; Reiser et al., 1995). The *bel1* mutant exhibits bell-shaped ovules – hence its name – caused by the abnormal development of integuments (Reiser et al., 1995). Occasionally, the *bel1 *mutant shows homeotic conversion of ovules into carpeloid structures due to prolonged *AG* expression during ovule development (Modrusan et al., 1994; Ray et al., 1994; Brambilla et al., 2007). BEL1 is required for auxin and CK signaling pathways during ovule development; the level and localization of *PIN1* expression are controlled by CK in part viaBEL1 activity (Bencivenga et al., 2012).

Inside the future fruit, tissues required for successful fertilization and fruit compartmentalization are formed concomitantly.Two placenta ridges develop in the medial plane to give rise to a specialized structure compartmentalizing the fruit, the septum, which divides the fruit into two halves. In its center, the transmitting tract differentiates in the apical-basal axis to guide pollen tube growth. To date, little is known about the role of *TALE* genes in septum development. SAWTOOTH1 (SAW1)/BLH2 and SAW2/BLH4, members of the BELL family, are expressed in the transmitting tract, and interact with STM and BP, but their exact role in medial tissue development remains to be determined (Kumar et al., 2007).

### POST-FERTILIZATION EVENTS

Upon fertilization, the gynoecium will develop into a fruit that contains the seeds. Gynoecium enlargement to accommodate the developing seeds relies on the coordinated growth of the entire organ, which strongly depends on hormonal balances (for review, Reyes-olalde et al., 2013). For instance, GA-deficient mutants show reduced fruit size, indicating that fruit development involves extensive GA-activated cell elongation (Koornneef and van der Veen, 1980; Chiang et al., 1995). While the fruit enlarges, differentiation processes take place to ensure efficient release of the seeds (Reyes-olalde et al., 2013). At the cellular level, this includes the differentiation of the dehiscence zone at the valve margins. This process depends on the activity of IND, which is responsible of the formation of a local auxin minimum at the valve margins through the regulation of *PINOID* and *WAG2 *kinases (Sorefan et al., 2009). The dehiscence zone consists of two cell layers: the lignified and the separation layers. The lignified layer, located at the boundary with the valve, is continuous with the lignified internal layer (endocarp b) and contributes to tension that builds up in the silique until dehiscence. The layer located on the replum side, which constitutes the separation layer, is composed of isodiametric cells that undergo middle lamella breakdown. This separation process involves the activity of specialized cell wall enzymes such as polygalacturonases (PGs) and pectin methylesterases (PMEs) that increase the ability of PGs to break down pectin (Ogawa et al., 2009 and for review, Wolf et al., 2009). A link between TALE proteins and cell wall modifications has been shown in studies of internode patterning in *rpl* and *bp* mutants (Mele et al., 2003; Smith and Hake, 2003; Peaucelle et al., 2011). BP prevents premature deposition of lignin during internode growth by direct repression of genes involved in lignin biosynthesis, and regulates other cell-wall-specific genes such as ones encoding PMEs or cellulose synthetase (Mele et al., 2003; Wang et al., 2006). RPL is involved in maintaining normal phyllotaxy via the regulation of PMEs, which are involved in the cell wall loosening necessary to allow growth (Peaucelle et al., 2011). Interestingly, *KNAT6* and *KNAT2*, which act antagonistically to *BP* and *RPL* in stems, are expressed in valve margins (Ragni et al., 2008). This is consistent with *KNAT6* expression in SAM and its role in maintaining boundaries between SAM and lateral organs (Belles-Boix et al., 2006). Inactivation of *KNAT6* rescues replum formation in *rpl* mutants, showing that the antagonistic interaction between *KNAT6* and *RPL* also controls fruit architecture. Consistent with their expression in valve margins, KNAT6, and KNAT2 positively regulate lignin deposition (Khan et al., 2012a, b). These factors also act antagonistically to BP during floral organ abscission, a process that also requires cell wall remodeling. *BP* regulates the timing of floral abscission by controlling abscission zone cell size*. *Upon activation of a signaling pathway including inflorescence deficient in abscission (IDA) and two receptor-like kinases, HAESA and HAESA-LIKE2 (HAE-HSL2), BP is inactivated, leading to an increase of *KNAT2* and *KNAT6* expression, which act as positive regulators of floral organ separation (Shi et al., 2011). The link between TALEs and cell wall remodeling enzymes was further confirmed with the identification of STM, KN1, and RPL targets, which include several genes involved in cell wall modifications (Spinelli et al., 2011; Bolduc et al., 2012; Etchells et al., 2012).

## FUTURE DIRECTIONS

Gynoecium development is critical for Angiosperm reproductive success, and is therefore tightly controlled by interconnected networks of TFs. Here, we reviewed the role of TALE TFs in the control of carpel development, and present the state of knowledge of the molecular interactions within this gene regulatory network. To date, the studies concerning the contribution of TALE TFs to carpel development focused on a few members of this family. Despite the number of studies, several pieces of the puzzle that will be needed to decipher the entire carpel regulatory network are still missing. In particular, the role of the KNAT class II members in carpels has not been investigated. Although several TALE members are expressed in carpels, the detailed expression pattern remains to be characterized for most of them. A precise map of TALE expression and co-localization of KNOX and BELL in the gynoecium will provide clues about putative partners and redundancies. Despite evidence linking TALE TFs, CK and GA pathways, the exact role of this regulatory node and its precise contribution to carpel development are not yet well established. Recently, the direct targets of KN1 in maize inflorescences were identified, and these data confirm that TALE TFs function as major orchestrators of hormone synthesis or response (Bolduc et al., 2012). Importantly, a clear link between KN1 and the auxin pathway was demonstrated. Furthermore, key developmental regulators such as homeodomain TFs are highly represented among KN1 targets, suggesting that KN1 orchestrates upper levels of regulatory networks controlling development. New strategies based on next generation sequencing to identify targets of TFs have begun to shed light on the molecular interactions downstream of key TFs, providing crucial insight into the mechanisms controlling development and opening new perspectives regarding carpel development. The integration of these data into comprehensive models accounting for spatial and temporal information represents a challenge to fully understand how fruits develop. Developing mathematical models will be particularly useful for understanding how fruit morphology can vary and how their astonishing diversity of shape can be achieved among plant species.

## Conflict of Interest Statement

The authors declare that the research was conducted in the absence of any commercial or financial relationships that could be construed as a potential conflict of interest.
